# How women manage recurrent urinary tract infections: an analysis of postings on a popular web forum

**DOI:** 10.1186/1471-2296-15-162

**Published:** 2014-09-26

**Authors:** Andrew Flower, Felicity L Bishop, George Lewith

**Affiliations:** Complementary and Integrated Medicine Research Unit, University of Southampton, Aldermoor Health Centre, Aldermoor Close, Southampton, SO16 5ST UK; Centre for Applications of Health Psychology, University of Southampton, Southampton, UK; Complementary and Integrated Medicine Research Unit, University of Southampton, Southampton, UK

## Abstract

**Background:**

Recurrent urinary tract infections (RUTIs) are commonly presented by women in primary care. In order to explore the poorly described experience of women with RUTIs a qualitative study was conducted that analysed data from a publically accessible internet-based self-help forum.

**Methods:**

Qualitative Description was used to analyse the text with an emphasis on using the naturalistic language of the informants to portray their perceptions and experiences of RUTIs. Individual codes were identified inductively and grouped according to common ideas into related categories, before being incorporated into five main themes.

**Results:**

Women of diverse ages and geographical location contributed to the website. Themes were identified that vividly explored the atypical symptomatology of RUTIs, the serious impact it had on many aspects of women’s lives, different attitudes to treatments options such as antibiotics, the use of unorthodox approaches such as complementary and alternative medicines (CAM) and contrasting experiences of medical practitioners.

**Conclusion:**

A web-based analysis can vividly capture the views of a diverse population. RUTIs can have a disabling effect on women’s health, their intimate and social relationships, self-esteem, and capacity for work. Further research is required to clarify the wider relevance of the qualitative themes identified, to identify key elements of good practice, and to provide a more rigorous assessment of CAM interventions.

## Background

In the UK urinary tract infections (UTIs) are the commonest bacterial infection presented by women in primary care [1] with approximately 40–50% of women experiencing one lifetime episode [2]. Recurrent urinary tract infections (RUTIs) are defined as three episodes of UTIs in the previous 12 months [3]. Between 20–30% of women who have one episode will have a further episode and around 25% of these will develop RUTI [4]. RUTIs can reduce quality of life and increase healthcare costs associated with outpatient visits, diagnostic tests and prescriptions.

Antibiotic prophylaxis can prevent RUTIs [5] but is commonly associated with unpleasant side effects such as oral and vaginal candidiasis and gastrointestinal disturbances, and occasionally more severe side effects. Once prophylaxis is discontinued, even after extended periods 50–60% of women will become re-infected within 3 months [6, 7]. In addition antibiotic overuse and the subsequent development of bacterial resistance is a growing problem [8] that increasingly affects management.

Although uncomplicated UTIs are considered a mild, self-limiting condition, qualitative research suggests that UTIs can seriously impact women’s quality of life [9–11]. Malterud and Baerheim [10] explored the symptomatic experiences of 94 Norwegian women with UTIs and reported an ‘unexpected finding’ of accompanying systemic symptoms including tiredness, inability to concentrate, and irritability. These systemic symptoms and the disruption they caused were also noted in qualitative interviews with 21 women in the UK experiencing acute UTIs [9]. The Norwegian study (10) also found that women used vivid language to describe their symptoms (such as “like peeing barbed wire”) that was more richly metaphorical and nuanced than the medical terminology of dysuria, urgency and frequency. Rink [12] found a similar disparity between the language used by UK GPs and 113 women with UTIs when describing risk factors for contracting an infection (for example GPs cited female anatomy as a prime risk factor for UTIs whilst this was not mentioned by any of the women involved in the study) and noted that this could have clinical implications. To our knowledge, there has been no qualitative study focussing specifically on the experiences of women with RUTIs and the impact that recurrent infection has on their lives.

In order to address this gap, a qualitative study was conducted that analysed naturalistic data available from an Internet forum dedicated to supporting women with cystitis. The selected web forum was hosted by The Cystitis and Overactive Bladder Foundation (COBF) [13], a support charity for people with bladder problems in the UK with 5,994 online members and an open access web-based message board forum with postings on 7,870 topics. This site is by far and away the largest and busiest UK online support community for women with bladder related problems and as such provides the greatest opportunity for a diverse range of experiences, perceptions and management strategies of women suffering from RUTIs.

## Methods

The methodological approach adopted for this analysis was Qualitative Description. This is an inductive method that uses the naturalistic language of the informants to portray their ‘perception and experience of the world and its phenomena’ [14] with an emphasis on precise low-inference description, rather than in-depth interpretation or development of an explanatory hypothesis. It is particularly suitable for ‘mapping the terrain’ of a poorly explored topic and gaining initial insight into informants’ views [15]. It can also incorporate quasi-statistical descriptive data, such as the number of times a posting has been viewed, to illustrate participant experience. Furthermore, when analysing web-forum postings it is not possible to probe participants for the very full and detailed narratives that would facilitate more in-depth qualitative analysis. For these reasons we considered Qualitative Description as an appropriate method for this study.

Text used for analysis was downloaded from the COBF website to Word and was then exported to the qualitative data management programme NVivo. The text was read and re-read multiple times, leading to the identification of individual codes that were identified inductively from the data. These were then grouped according to common ideas into related categories, and finally incorporated into five overarching main themes that are presented in this paper. One researcher led the analysis (AF). Coding and identification of themes were discussed in detail with a second researcher (FB) who was also familiar with the raw data and again among the whole research team to avoid idiosyncratic or selective interpretations and offer diverse perspectives. Using NVivo facilitated the maintenance of a detailed audit trail. Initially the ‘Meet and Greet’ section of the cystitis section of this web forum was analysed because it introduced many of the key issues developed throughout the site and provided background information on many of the women who use the forum. The remainder of the site, covering (at the point of analysis) 915 separate topics specifically relating to bladder infection comprising of 5,386 postings (see Table 1), was then analysed to provide a more detailed evaluation, looking for contradictory viewpoints, and to investigate areas not discussed in ‘Meet and Greet’.

**Table 1:** **A sample of topics on p1 (of 40) on the COBF Cystitis forum**

Subject	Author	Views	Replies	Last post
Meet and greet 1 2 3 4 5 6 … last page		29,691	148	May 10 by
Cystitis success stories		3,806	9	April 16, 2011 by
Cytoscopy…good idea?		138	5	June 30 by
cystitis and the pill		125	2	June 26 by
Biofilms and chronic infection		190	0	June 10 by
All UTI’s symptoms but no bacteria found		4,437	8	May 30 by
Wild oil of oregano - anyone else tried this?		228	2	May 24 by
After effects of antibiotics?		203	2	May 23 by
Any success without antibiotics?		398	4	May 22 by
Antibiotic after sex?		317	2	May 21 by
hi everyone - my cystitis story 1 2		986	19	May 21 by

Permission was sought from the COBF and from Southampton University Ethics committee to analyse data that was publicly available on the website. This was granted in February 2012 (University of Southampton Ethics and Governance Online ID 946) and analysis of all material posted on the site from its inception in 2004 began in the same month and was completed in June 2012.

In recent years there has been an ‘exponential’ increase [16] in the use of Internet websites to provide health related information and to operate as forums where site users can share experiences, discuss treatment options, and gain support. There has been a parallel increase in web-based research to analyse these data and many studies now report on the content, form, and function of these sites for a wide range of health related conditions [17–19]. Web-based research into the experience of an illness such as RUTIs has the advantage of enabling access to many women from a wide geographical spread and diverse socio-economic backgrounds with 80% of UK households having internet access in 2012 [20]. Ecological validity is enhanced because material reflects women’s spontaneous communication in their own words about their experiences. An internet forum also allows participants to preserve their anonymity, which may make participants more comfortable discussing personal, sensitive issues [21].

The main ethical issues related to this research revolved around the use of women’s responses on a web forum without their explicit permission [22]. We have adopted the recommendation for an inductive, casuistic approach to these issues that takes into account the specific context of the women involved and the site being investigated, and balances the potential harms and benefits that could result from this research [23, 24]. Consequently data has been drawn only from the publicly accessible forum and not from any password protected, private chat rooms that women could choose to use on the same site. As a result women participating on the site can be considered as providing their tacit consent for open access to these discussions [23, 25]. To help preserve their anonymity the names of women quoted in the paper have been changed. Finally, with the permission of the site managers, a version of this paper and an accompanying letter was posted on the COBF website explaining the rationale, methodology and purpose of the research. The letter clearly gave site users the option of withdrawing any comments they had posted from use in this paper. One site user did make contact and gave her permission for her material to be used as long as it was done so anonymously.

Legally the copyright of the text belongs to the COBF and it is clearly stated that ‘The COB Foundation is the owner or the licensee of all intellectual property rights in our site and the material published on it’. Permission was requested from the COBF and granted prior to commencing this project.

We believe that this research conforms to the recent ethical recommendations for Internet mediated research outlined by the Association of Internet Researchers [23] and the British Psychological Society [26]. It is respectful of the women who contributed to the forum, and uses a scientifically valid methodology to present and analyse the findings. It is socially responsible, and may contribute to improving the future provision of care for this group of women. For these reasons we feel that this web-research project is ethically justified.

## Results

### A range of participant experience

‘*I am 43 yrs old and have been experiencing cystitis type problems for 25 yrs.*’ *(Abby)*.

Visitors to the site who disclosed their ages range from 13 to 65 years old and, although most women live in the UK, women from Ecuador, Canada and Australia have contributed to the discussion. The duration of disease ranges from a year to several decades. Frequency and severity of symptoms also vary considerably with some women reporting ‘*a serious attack about once every two or three months*’ (Beth) compared to others who suffer from ‘*constant pain*’ (Cara). Many of the women report a progressively deteriorating illness with increasing frequency of symptoms.

Site users often describe a long history of recurrent UTIs. Most have received many years of conventional intervention, mainly antibiotics delivered as short or long term prophylaxis. Cystitis is commonly associated with sexual activity but several women report a history of UTIs that stretches back to early infancy. By contrast another group of older women report symptoms beginning with or being aggravated by menopausal hormonal changes.

The following key themes emerged from an analysis of the website.

### Symptoms that don’t live in the textbooks…

‘*It’s so depressing - I don’t get ‘simply cystitis- that infectioin (sic) that seems to only live on in text books!’ - I get vile infections that are really hard to shift. I get shaking and shivering, terrible diarrhea, waves of nausea and generally end up losing about half a stone when I get an attack*’ (Dora).

Women described far broader, more diverse, systemic, and disabling symptoms than those described in the classical medical literature, i.e. frequency, urgency, burning and lower abdominal pain. These include feeling ill and having persistent widespread discomfort:

‘*I’m feeling quite ‘fluey’ at the moment, and having spasms around my waist and up my back, there is a ‘knot’ above my pubic bone in my bladder and the pelvic floor doesn’t feel right at all. My legs are killing me, and there is also some stabbing in my right hip*’ (Eva).

Notions of causality also differ considerably from the standard medical model. Several women, in the absence of cultured evidence of bacteraemia, subscribe to little understood, non-pathogenic (according to current understanding) bacteria such as ureaplasma, as the cause. Some women associate their cystitis with acute or prolonged episodes of stress, lack of self-esteem, or ‘guilt/shame’: ‘*my chronic BC was definitely triggered by my emotions….. Once I got to grips with the effects of negativity from my past the infections stopped*’ (Fiona). Women understood these emotional influences as being mediated via damage to the immune system or via rather nebulous concepts of ‘inflammatory hormones’.

### ‘I’m finding this affects every aspect of my life’ (Emma)

For women using this website it is apparent that RUTIs have a far greater impact on the quality of their lives than is commonly acknowledged in the medical literature. Even relatively mild symptoms of frequency and urgency can disrupt sleep, create anxiety, and lead to persistent fatigue. Knowing that intercourse can be a trigger for an infection can undermine intimate relationships: ‘*It (cystitis) only ever happens after intercourse and has left me terrified to have sex, this illness has ruined numerous relationships for me*’ (Gabby). The impact of cystitis on sexual relations is a much-discussed theme on the site. In total 73 women have made over 122 postings to the discussion ‘cystitis and sex’, which has received over 36,342 viewings and is the most commonly viewed topic within the forum. Those who maintain sexual relationships with the background threat of UTIs, report on their reluctance to have intercourse and describe how negative associations can be made, with infection leading to one woman being ‘*quite disgusted with sex’* (Harriet). Even with an understanding partner, this can threaten long-standing relationships: ‘*My husband is very understanding, but I’m extremely depressed by the fact that we can no longer be intimate without weeks of pain afterwards*’ (Issy).

RUTIs can also disrupt other areas of a woman’s life with many reports of enforced periods off work with financial and social consequences: ‘*The UTIs have taken a huge toll on my sex-life (and therefore my relationship), work, social life and finances* (Abby)’.

For those who are not in paid work the impact can be equally traumatic: ‘*I am a mother of four, and currently have no interests or hobbies due to being in so much pain all the time*’ (Kate).

The prospect of a UTI also creates anxiety about future plans, with women expressing their concerns about being able to attend a daughter’s wedding, to go on holiday, or to enjoy an approaching honeymoon. It is hardly surprising then when Lizzie exclaims: ‘*I just want to be normal again*!’

### Resisting antibiotics

‘*Each time I have come off the a/bx the symptoms return*’ (Maria).

Many of the women using the site described extensive experience of antibiotic use. There are 37 separate topics with a heading relating to antibiotics on the forum. Resistance to these drugs appears fairly commonplace among these women, as Naomi describes:

‘*Over the years I have used many different antibiotics, some times to clear infections and for a period of about 6 years I took them when we had sex as a preventative. However I am now resistant to many of these drugs*’.

Even when the drugs work symptoms often return fairly immediately:

‘*Antibiotics dont seem to help much now, helps a little at the start but seems to come back just as quick when its chronic, and also now more reluctant to take long term antibiotics*’ (Olive).

When antibiotics are successful they can be transformative as Jenny expresses: ‘*long term antibiotics have allowed me to get my life back on track*’. However for those women whose symptoms respond to antibiotics there is considerable anxiety about the prospect of resistance developing; ‘*I also worry that I will become resistant to this too and then I will be left without an antibiotic which works for me*’ (Petra).

On the website, attitudes to antibiotics range from disregarding them in favour of alternative remedies to a more conventional view insisting on the need for antibiotics as a way of preventing a more serious kidney infection.

Women express a number of concerns about antibiotics including anxiety over side effects, fear of them not working, anger both over their being ‘dispensed like sweets’ or in relation to their perceived role in aggravating long term bladder conditions. Anxieties about side effects include immediate concerns about nausea, thrush, and diarrhoea. More long term concerns centre around the potential impact of antibiotics on the immune system: ‘*I spent months on antibiotics, which I think knocked my immune system even more, and made it easier for the next infection to come along!*’ (Rachel). Thus some women perceived a negative cycle in which an infection triggers antibiotics, which provide temporary relief but then make them more likely to contract another infection.

Several site users report on conventional treatments used in addition to antibiotics. Urethral stretches are the commonest of these interventions. Some women report positive responses: ‘*I had a cystoscopy and a urethral stretch… I do still suffer from recurrent cystitis but I have to say it hasn’t been anywhere near as severe as any of the bouts I had in the past*’ (Susan). However other women report short-term benefits that do not stand the test of time.

### Seeking alternatives…

‘*Does anyone else out there have any other, herbal or alternative therapies*’ (Olive).

Concerns over effectiveness and adverse effects mean women on the forum show considerable interest in other conventional treatments and in Complementary and Alternative Medicines (CAM) including dietary and lifestyle changes and acupuncture and herbal medicines. Typically women may use several different therapeutic modes that are combined into a complex CAM intervention, as exemplified by Rachel’s approach:

‘*I’ve been seeing an acupuncturist since September, who’s helped a great deal, especially with the pain and frequency. I’ve also seen a nutritionist, who put me on a diet with lots of supplements, including large doses of vitamin C and cranberry tablets, which really helped make me stronger. I’ve also found washing with tea tree oil helps. I have to avoid spicy food, caffeine, sweet fizzy drinks and most alcohol*’.

Within the forum there is considerable diversity of views expressed about CAM. For some women CAM therapies have not been helpful:

*I have tried pretty much every alternative thing under the sun:waterfall d-mannose, reflexology, homeopathy (inc the tincture you mention), various “miracle promising” supplements. The only thing that has been remotely successful for me has been a long term course of antibiotics*’ (Vicky).

Women described using CAM interventions in conjunction with conventional antibiotic treatment (e.g. to alleviate the side effects of antibiotics, or by ‘*strengthening the immune system*’ to help prevent relapse) or as an alternative anti-bacterial approach (eg herbs such as uva ursi or cranberry). Incorporating CAM appears to offer some women a greater sense of control and empowerment, and led them to consider the possibility of a deep and permanent ‘healing’ to address the multifaceted aspects of RUTIs, as opposed to temporary disease suppression:

‘*The key is to get the body to heal properly, that takes alot of time and alot of focus. It is layers of healing - so sorting out IBS, the bladder, the gut, it all takes a long time to re-balance. Using alternative therapies mean that you feel more confident when twinges come on and you feel more in control, as you know you can treat them herbally and you don’t have to take anti-biotics*’ (Wanda).

### Doctors: heroes and villains

‘*A urologist a few years ago (I had no faith in him at all) asked me about my hygiene, I felt like hitting him!!*’ (Yasmine).

Women expressed quite polarised views towards their doctors. Some describe them as ‘unsupportive’ and ‘dismissive’, often ineffective and at worst unsympathetic and uncaring. ‘*my doctor’s useless as well, he just sais (sic) “its normal for women to get these infections” well what use is that to me*’ (Hilary). Other women describe a lack of care and understanding of the severity of their experience that may be exacerbated by gender differences between patient and practitioner: ‘*I had had a bad experience in the past of a GP who refused to prescribe me anything until he (of course it was a man!) had sent off a urine test. This would’ve meant waiting days for the results and I was in agony*’ (Sue).

In some instances doctors were seen as being impractical and patronising:

‘*I only wish the doctor had given me specific tips rather than the ‘try to keep clean’ nonsense which left me feeling down*’ (Eva).

Many women describe frustration and dissatisfaction at what they regard as inadequate pattern of care.

This pattern of poor practice is exemplified by Linda’s description of her encounter with a poorly informed, apparently unsympathetic GP, who is perceived as inattentive and patronising. The prescription of an inappropriate course of antibiotic treatment and a rather dismissive attitude to her condition leaves Linda frustrated at what she regards as inadequate treatment for a potentially serious illness:

‘*I finally managed to get another appointment at my doctors today. Last week I had to go into the walk in medical centre as I had a urine infection and couldn’t get a doctors appointment. They gave me a 3 day course of cephalexin which made me feel much better*’.

*Today I asked my own doctor for some preventative antibiotics as I have just had an infection from not having them and he refused. He gave me a week’s course of trimethoprim to clear up the infection he said. I said I had already been treated for that but he just ignored me. When I got home I found out I can’t take them anyway as I am breastfeeding and those ones pass into the milk in high doses. He didn’t even look at my urine test results that were sent away by the walk in medical centre and didn’t even test my urine*.

*I asked to be referred to a urologist again and he said “Why do you want to see one? They will only put you on a preventative dose of antibiotics”. I told him that I have had urinary problems since I was 8 years old and I am in agony with a urethral stricture and my bladder is permanantly full plus I have interstitial cystitis. He looked at me as if I was a hypercondriac. I had to practically beg him until he finally agreed to refer me. He said there is a massive waiting list though and I could be waiting over a year*.

*I feel like screaming in frustration as the doctor won’t listen to me. I know my body better than anyone and know how to treat it but I just get treated like a timewaster and told to drink plenty of water. He even said that an infection will go on it’s own accord if I drink enough. Well if he bothered to read my notes he would see that I had to have kidney scans a few years ago after a really bad infection that lasted 8 weeks as my old doctor was worried my kidneys may have been damaged*’.

By contrast some women reported having a ‘great’ or ‘brilliant’ doctor who was conscientious, caring and supportive-even though not always to great effect: ‘*my GP is brilliant, have had every test done imaginable and taken loads of antibiotics which relieve but doesn’t get rid of the pain*’ (Elena).

Reports of a positive interaction with GPs repeatedly emphasise a woman’s relief (and often surprise) to find that their doctor listened and was responsive to their complaint, that they had read the notes and were informed about the particular presentation of the woman and RUTIs in general. They demonstrated understanding and kindness and were willing to refer to more specialist expertise.

*‘Now I am with a doctor with a very specific interest in women’s urology, their team is so sympathetic and immediately recognised that an inability to have sex is a problem which shouldn’t just be accepted as something you have to live with, they also really understood the emotional and psychological impact that it has on those of us who have this problem’* (Libby).

## Discussion

A web-based analysis has the advantage of capturing the views of a large and diverse population of women with a shared condition. Many of the accounts on the web forum are deeply personal, detailed, and moving. Women have articulated their experience both for themselves and for other site users. Dialogues frequently develop and many women express feelings of relief at finding people with similar experiences who understand and sympathise with their condition. The level of disclosure found on the site concurs with previous findings that the use of an internet forum has a disinhibiting effect and encourages a degree of openness and honesty that may be difficult to replicate in face-to-face discussion [19].

RUTIs are a relatively common but often poorly managed condition that can cause distressing local and systemic symptoms. Although frequently described in medical parlance as being a minor, self-limiting condition, for many women RUTIs cause severe physical discomfort and are associated with significant psychological distress, damaged relationships, and an inability to work or socialise that seriously undermine the quality of their lives. These reports are consistent with previous research describing wide ranging and frequently atypical symptoms of acute episodes of cystitis [10, 11] that can adversely affect psychological wellbeing, social activity, and quality of life [9, 11].

Site-users’ experiences of medical treatments are complex and diverse. Some women report frustration at what they regard as dismissive, patronising and uncaring treatment and express dissatisfaction with the side effects and short-term benefits of antibiotics. As a consequence Complementary therapies are widely used by women on the forum as primary or adjuvant treatments to alleviate symptoms and to address the perceived deeper immunological or emotional causes of these infections.

However women also report encountering excellent physicians and receiving sustained benefits from their treatment. Although the qualities of good doctoring may appear self-evident it is still both useful and salutary to have these explicitly identified by women who are clearly immensely relieved to be taken seriously, listened to, and cared for by well-informed physicians.

These contrasting findings support a recent analysis of older women’s reports of receiving a similar mixture of adequate and inadequate conventional treatment for UTIs [11]. In our account the medium of a web forum allows a particularly vivid articulation of the frustration over poor care and ineffective treatment. In part this may be a result of the observed phenomena that people interviewed via the Internet find it easier to register protest in the absence of a face-to-face interviewer [27]. It may also be due to the constant availability of a web forum where users can post comments in ‘real-time’, such as on their return from a difficult GP consultation, or in the middle of the night when they are in pain and unable to sleep. These immediate and often quite raw reports are captured ‘on line’ and are given extra poignancy and significance as they become part of a narrative sequence lasting over several months and even years.

There are several limitations to this analysis. Participation in the forum was contingent upon having access to online computers or phones and possessing the skills, confidence, and desire to engage with online facilities. Women using the site were a self-selected sample who became actively involved with the COBF often because their condition was not well managed. This may mean that they had more extreme views and more difficult experiences than other women with RUTIs who did not post on the COBF forum. Therefore reported findings should only be very cautiously extended to the wider population of women suffering from RUTIs. There are also limitations in the data itself. Conducting a retrospective text-based analysis means that it has not been possible to ask participants to expand on or clarify their postings. Face-to-face interviews would have permitted a more detailed, responsive, and nuanced investigation of a woman’s experience. Also the emphasis on this study has been on the content generally with a particular focus on a few specific themes. This was consistent with the study aims, to explore women’s experiences of and perspectives on RUTIs, as represented on a major web-based forum. Analyses of the development of website narratives or of the diverse functions of the site for its users, for example, have not been conducted, but would be interesting areas for future investigation. Considerable more work needs to be done into how the medium of a web forum influences the way in which people can articulate and represent their life experiences.

## Conclusion

This research into RUTIs appears to be the first qualitative investigation of this neglected area. It confirms the findings of previous studies [9–11] that UTI occurrence can have a systemic impact on health and wellbeing and cause serious disruption to daily activities, and illustrates how the recurrent nature of these episodes amplifies and compounds this disruption into a serious long-term disability. The women’s use of metaphor captures their experience more effectively than the more reductive terminology used in medical descriptions. The use of Qualitative Description allows these voices to be vividly heard and enables the presentation of a diverse picture of what it means for some women to live with the uncertainty and discomfort of recurrent UTIs.

For some forum users, the judicious use of antibiotics, together with sympathetic and informed doctoring, appears to provide some relief. However others continue to suffer physically, emotionally and socially from RUTIs. Further research of women’s experience of this troublesome condition is required to clarify the wider relevance of the qualitative themes identified here, to identify key elements of good clinical practice and supportive care, and to provide a more rigorous assessment of alternatives to conventional treatments such as CAM interventions.

## Authors’ information

Andrew Flower is currently funded as a NIHR Post doctoral Fellow investigating the possible role of Chinese herbal medicines in the treatment of recurrent urinary tract infections.
